#  Beyond the lab ? Eye tracking in dynamic real-world environments 

**DOI:** 10.16910/jemr.12.7.2

**Published:** 2019-11-25

**Authors:** Enkelejda Kasneci

**Affiliations:** Informatik, Universität Tübingen, Germany

**Keywords:** eye tracking, real-world environment

## Abstract

Keynote by Enkelejda Kasneci at the 20th European Conference on Eye Movement Research (ECEM) in Alicante, 18.8.2019,

**Video stream** of presentation: https://vimeo.com/356314274

